# Migraine Comorbidities

**DOI:** 10.3390/life14010074

**Published:** 2024-01-02

**Authors:** Dan Iulian Cuciureanu, Cătălina Elena Bistriceanu, Georgiana-Anca Vulpoi, Tudor Cuciureanu, Florina Antochi, Adina-Maria Roceanu

**Affiliations:** 1 Neurology Department, Faculty of Medicine, “Grigore T. Popa” University of Medicine and Pharmacy, 700115 Iasi, Romania; cuciureanudan@yahoo.com; 2 Neurology Department I, “Prof. Dr. N. Oblu” Emergency Clinical Hospital, 700309 Iasi, Romania; vulpoi.anca@yahoo.com; 3 Elytis Hospital Hope, 700010 Iasi, Romania; 4 Gastroenterology Department, Faculty of Medicine, University of Medicine and Pharmacy, 700115 Iasi, Romania; drcuciureanutudor@gmail.com; 5 Neurology Department, University Emergency Hospital, 050098 Bucharest, Romania; flrant@yahoo.com (F.A.); amr2012mar@gmail.com (A.-M.R.)

**Keywords:** migraine, comorbidities, epilepsy, anxiety and mood disorders, CGRP, gut–brain axis, microbiome

## Abstract

Novel knowledge about the interrelationships and reciprocal effects of migraine and epilepsy, migraine and mood disorders, or migraine and irritable bowel syndrome has emerged in recent decades. Over time, comorbid pathologies associated with migraine that share common physiopathological mechanisms were studied. Among these studied pathologies is epilepsy, a disorder with common ion channel dysfunctions as well as dysfunctions in glutamatergic transmission. A high degree of neuronal excitement and ion channel abnormalities are associated with epilepsy and migraine and antiepileptic drugs are useful in treating both disorders. The coexistence of epilepsy and migraine may occur independently in the same individual or the two may be causally connected. The relationship between cortical spreading depression (CSD) and epileptic foci has been suggested by basic and clinical neuroscience research. The most relevant psychiatric comorbidities associated with migraine are anxiety and mood disorders, which influence its clinical course, treatment response, and clinical outcome. The association between migraine and major depressive disorder can be explained by a robust molecular genetic background. In addition to its role as a potent vasodilator, CGRP is also involved in the transmission of nociception, a phenomenon inevitably linked with the stress and anxiety caused by frequent migraine attacks. Another aspect is the role of gut microbiome in migraine’s pathology and the gut–brain axis involvement. Irritable bowel syndrome patients are more likely to suffer migraines, according to other studies. There is no precise explanation for how the gut microbiota contributes to neurological disorders in general and migraines in particular. This study aims to show that migraines and comorbid conditions, such as epilepsy, microbiota, or mood disorders, can be connected from the bench to the bedside. It is likely that these comorbid migraine conditions with common pathophysiological mechanisms will have a significant impact on best treatment choices and may provide clues for future treatment options.

## 1. Introduction

Migraine is a primary headache, with a high prevalence and morbidity among young adults, especially women, that significantly reduces the quality of life [1,2]. Approximately 1 billion people worldwide suffer from migraine and this disorder remains the second leading cause of disability worldwide, after low back pain, despite considerable progress in diagnosis and treatment [3,4]. The incidence of migraine attacks peaks between early and mid-adolescence, although attacks can occur at any age. One-year migraine prevalence is 11 percent in the United States and Western Europe, 6 percent in men, and 15 to 18 percent in women [5,6,7]. Women are three times more likely to suffer migraines than men (gender ratio: 3:1), and migraines usually occur between ages 25 and 55 [8,9].

In this article, we review migraine pathophysiology and new theories regarding migraine comorbidities.

## 2. Common Types and Red Flags

Several types of migraine are recognized by the International Headache Society in 2018, among them migraine without aura and migraine with aura.

Previously known as common migraine, migraine without aura is characterized by recurrent headaches with specific characteristics [10]. A migraine attack usually lasts 4–72 h. It is frequently unilateral, pulsating, mild to severe in intensity, and accompanied by vegetative symptoms, photophobia, and phonophobia [11].

In migraine with aura, headache is accompanied by or preceded by transient focal neurological symptoms [10]. Visual, sensory, and language disturbances are typical migraine aura symptoms [12]. The most common form of aura in migraine with aura is a visual aura, which occurs alone or in combination with other auras in 98% of cases [13].

There are many pathologies that may mimic primary headaches. The following are signs that the headache may be a sign of an underlying disorder: new onset headaches, change in previously stable headache patterns, sudden peaking headaches, headaches that change with posture, headaches that awaken the patient, or headaches triggered by physical activity, the onset of first headache episode ≥50 years of age. In migraines with persistent auras on one side or brainstem aura, brain MRI is recommended [14].

If red flags are found during the anamnesis and physical examination, the Headache Consortium Guidelines recommends investigations for patients. The probability of significant intracranial pathology in a migraine patient with no red flags and a normal physical exam is only 0.18% [15].

## 3. Migraine Pathophysiology

Two main classical theories are described in the current literature: the vascular theory and the central neuronal theory. These assume complex peripheral and central processes involved in migraine pathophysiology [16]. Despite incomplete understanding of migraine’s pathogenesis, it is believed to involve the trigeminal nerve, which projects in axonal directions into the intracranial vasculature (trigeminovascular system) [17].

A study conducted by Wolff in the late 1930′s found dilated extracranial (temporal) arteries in migraine sufferers. Vasoconstrictors (ergotamine) relieved the pain and decreased arterial dilation in these patients [18,19]. According to Wolff’s vascular theory, migraine aura is caused by intracranial vasospasm of the cerebral arteries, while the pain is caused by extracranial vasodilation (together with a lowered pain threshold, intramural vascular edema and local sterile inflammation) [20].

Olesen and his research group demonstrated that the pattern of spreading oligemia (and, presumably, cortical spread depression) did not conform to the anatomical boundaries of the major cerebral blood vessels by measuring regional cerebral blood flow during migraine aura. The first part of Wolff’s theory was discredited, but there was no evidence for the second part concerning extracranial vessels [20,21].

However, migraine is considered a neurovascular headache, meaning that neural processes result in vasodilation [22]. The trigeminovascular system is one of the key factors in migraine pathophysiology [17]. In 1984, Moskowitz premised that the pain transmission in migraine originates in activation of trigeminovascular neurons that innervate meninges and their vessels [17,23]. The process implies activation and sensitization of these neurons.

**Figure 1:**
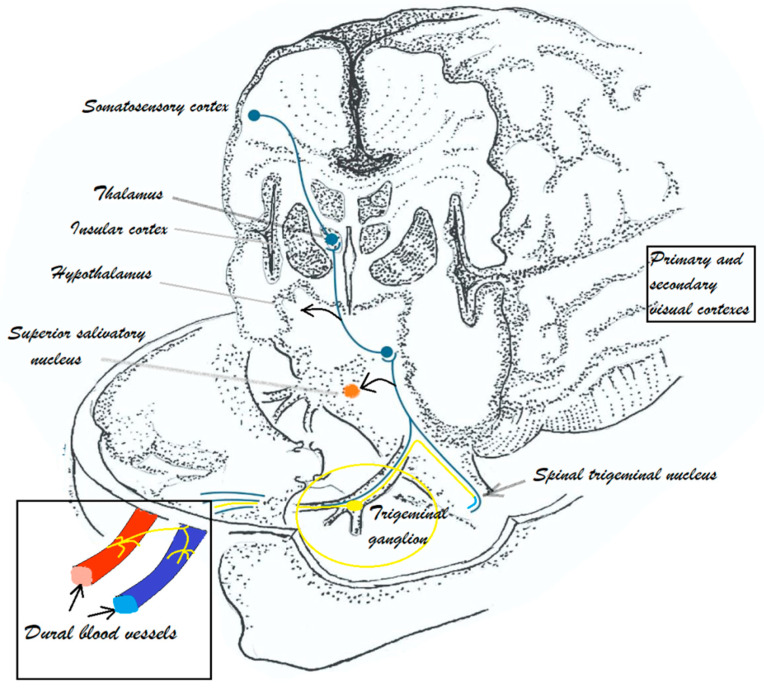
Trigeminovascular system in migraine; brainstem and cortical networks involved in pain and associated symptoms in migraine (Drawn by Diana Laura Blăjuță, Annotation by Cătălina Elena Bistriceanu).

In Figure 1 the nociceptive transmission from first-order trigeminovascular neurons (afferent fibers that innervate meninges and its vessels, cell body in trigeminal ganglion) to second-order trigeminovascular neurons (spinal trigeminal nucleus) and third-order trigeminovascular neurons (thalamus) is described; it is then transmitted to the somatosensory cortex and other cortical areas, ultimately resulting in pain perception. Based on preclinical data, activating the trigeminovascular system leads to parasympathetic outflow from the superior salivatory nucleus (SSN) and sphenopalatine ganglion to intracranial arteries. The release of pituitary adenylate cyclase activating peptides (PACAP), vasoactive intestinal polypeptides, and nitric oxide contribute to intracranial artery dilation [16].

In recent years, the central sensitization hypothesis has been proposed to explain many of the temporal and symptomatic aspects of migraine, as well as the poor response to triptan therapy when it is initiated hours after pain has started. The hypothesis proposes that altered sensory input processing in the brainstem, particularly in the trigeminal nucleus caudalis, may be responsible for migraine [24].

Despite incomplete understanding of migraine’s biochemistry, signaling pathways that may lead to migraine attacks have been identified; preclinical studies have shown that activation of the trigeminovascular system releases an antidromic signaling molecule calcitonin gene-related peptide (CGRP) [17,25]. CGRP is released by the C-fibers of the trigeminal ganglion and peripheral trigeminal nerve terminals as a result of neurogenic inflammation [26].

It has been shown in multiple studies that migraine attacks cause an increase in the release of the CGRP [27]. Additionally, neuropeptides found in nerves that supply extracerebral vessels, such as vasoactive intestinal polypeptide, substance P, and neurokinin A, are important vasodilators. As compared to other neuropeptides associated with the trigeminal and autonomic nerves, CGRP levels were elevated in external jugular vein blood samples during headaches [28]. The release of CGRP is controlled by presynaptic receptors on trigeminal neurons and the calcium-dependent exocytosis of CGRP occurs during nerve stimulation or depolarization [29].

The CGRP pathway is better understood with the help of current migraine treatments. 5-hydroxytryptamine (5-HT) 5-HT1B and 5-HT1D agonists such as triptans and ditans have been shown to suppress CGRP release from trigeminal presynaptic neurons during migraine attacks [30].

CGRP antagonists are medications that block the CGRP receptor. Recent CGRP antagonists can be divided into two main types: monoclonal antibodies and non-peptide small molecules, also known as gepants [31].

At the trigeminal ganglion, trigeminal nerve fibers, and dura, gepants (and mAbs targeting the CGRP pathway) seem to block pain amplification and perpetuation processes; they disrupt CGRP-mediated pain signals occurring via neuroglial, neuronal, and neurovascular pathways [27,32].

Antibodies targeting the CGRP pathway do not cross the blood–brain barrier, indicating that these drugs act on the peripheral segment of the trigeminovascular system [32].

Anti-CGRP receptor monoclonal antibodies are widely considered to be one of the most promising migraine prevention drugs, which may particularly be useful for chronic migraine patients who have resisted therapy. In order to prevent migraine attacks, these monoclonal antibodies represent the first mechanism-based, disease-specific treatment for migraine prevention. Compared to other migraine preventive medications, targeted molecules are more specific [33].

## 4. Epilepsy and Migraine

Over time, there has been an association between epilepsy and migraine. This means that seizures can trigger migraines and that migraine attacks could have an epileptogenic component [34]. Migraine is estimated to be 8–24% prevalent in populations with epilepsy, which means that migraine risk is approximately twice as high as in the general population. At the same time, people with migraine have a higher incidence of epilepsy (range 1–17%) than the general population (0.5–1%) [34,35,36,37].

In 2004, the International Headache Society proposed the criteria for migralepsy: 1—criteria for migraine with aura and 2—a seizure that occurs during or within 1 h after a migraine aura. However, few cases have been described in the literature and the relationship between these two has not been clarified [37].

The underlying mechanisms that trigger migraine attacks have been studied over time, and similarities have been found between them and those that trigger seizures with focal onset. The phenomenon of cortical spreading depression (CSD) was studied with functional imaging in people that experience migraine aura and transgenic animal models of familial hemiplegic migraine. There is a characteristic feature of CSD in migraine: a depolarization block after hyperexcitability that induces long lasting neuronal depression. Similarly, depolarization block is observed in ictal, interictal, and postictal epileptic activity [38].

CSD causes trigeminal-vascular system activation, with subsequent vasoactive peptides releasing in leptomeningeal space. These peptides (CGRP, substance P) induce vasodilation and sterile inflammation with pain [39].

After convulsions and after cortical spread depression, which is the neuronal mechanism of the migraine aura, there is an increase in extracellular glutamate, an excitatory neurotransmitter. After CSD, the trigeminal nucleus is involved with consequent central sensitization and pain; headaches and migraine aura may trigger seizures in epilepsy patients [40].

Glutamate is also an excitatory neurotransmitter implicated in aberrant signaling from epilepsy. Extracellular glutamate increases after seizures and has excitotoxic effects [41].

In everyday practice, visual symptoms associated with occipital seizures are sometimes mistaken for visual auras in migraine, despite the fact that they tend to display typical characteristics that distinguish them from migraine. Although EEG studies are not useful for routine headache evaluation, 24 h video-EEG studies in isolated cases with migralepsy showed EEG changes not typical of epilepsy during the migraine aura; moreover, epileptic ictal headaches do not have a specific pattern [39].

Several gene mutations associated with familial hemiplegic migraine type 1 (FHM1) and FHM 3 have been described. In familial hemiplegic migraine 1 (FHM 1), there are genes that encode P/Q calcium channels in FHM 3 for voltage-gated sodium channels. These mutations are likely to be responsible for an increase in neuronal glutamate [42]. There are *SCN1A* mutations with both FHM and epilepsy, like L263Q, T1174S, Q1489H, and L263V. As a result of the L263V mutation, sodium channel inactivation is accelerated, thus prolonging the duration of action and increasing neuronal excitability. A patient with this gene mutation may have epilepsy and FHM3 [43,44].

Another studied gene is *CACNA1A* that encodes a subunit of the P/Q calcium channel. Its mutation may damage calcium channel function and cause generalized epilepsy or FHM [34,44].

Apart from genes in FHM that encodes ion channels, there are genes such as *ATP1A2* that regulate membrane potential and inhibit Na⁺/K⁺ ATP-ase. As a result, migraine seems to be fundamentally a disorder of altered neuronal excitability in a similar way to epilepsy [44,45].

Another link between migraine and epilepsy is that anti-seizure drugs (ASD) can prevent migraine attacks. Perhaps this treatment involves peripheral dural or central trigemino-vascular mechanisms. Studies on animals have shown an inhibition of neuronal firing in the trigeminocervical complex and modulation in trigeminovascular transmission after intravenous topiramate [46,47]. Topiramate also inhibits the CGRP secretion from activated trigeminal neurons and may target cortical spread depression [48].

Another ASD used in migraine prophylaxis is valproate through GABA activity enhancement. In this way, there is a suppression of migraine events from the cortex, perivascular parasympathetic, and trigeminal nucleus caudalis [49].

There are also other studies of ASD in migraine prophylaxis like levetiracetam, lamotrigine, and gabapentin. Levetiracetam has a modulatory effect on GABAergic system and one study with the help of magnetic resonance spectroscopy proved a low GABA level in posterior cingulate cortex in persons with migraine that received levetiracetam monotherapy [50,51].

According to some authors, there is no direct cause-and-effect link between migraine and epilepsy. There is a high probability that they are both caused by overexcitation of cortical neurons. In epilepsy, cortical hyperexcitability leads to abnormal hypersynchronous electrical discharges, while in migraine, this hyperexcitability leads to cortical spread depression. In epilepsy, hyperexcitability alters ion exchange processes across membranes, leading to recurrent seizures, whereas in migraine, extracellular glutamate concentrations rise, resulting in an efflux of K+ ions, further affecting neuronal activity and resulting in brain hyperactivity [52].

Patients with both migraine and epilepsy experience symptoms sporadically and the interictal interval between symptoms varies. An alteration in the structure of ion channels can lead to an influx of pathological signals, such as seizures and migraines, to overcome homeostatic mechanisms. In epilepsy, a-amino-3-hydroxy-5-methyl-4-isoxazolepropionic acid (AMPA) receptors mediate seizure generation and spreading; in migraine, N-methyl-D-aspartate (NMDA) receptors are involved in CSD phenomenon [34].

During a study conducted on 597 patients with epilepsy, migraine and seizure-related headache were studied in this group. Prodromal and postictal seizure-related headache occurred more frequently in patients with migraine than those without and it was more often a migraine-type. According to the study, migraine is an important comorbidity of epilepsy, affecting the severity and characteristics of seizure-related headaches [53].

## 5. Depression, Anxiety and Migraine

Anxiety and depression are two of the most prevalent comorbid conditions associated with migraine, impacting disease prognosis, treatment, and clinical outcome. Researchers have found that people with migraines are five times more likely to develop first-onset major depression than those without migraines. At the same time, the risk of first-onset migraine is three times higher for people with a lifetime depressive disorder than for those without it [54].

The likelihood of suffering from anxiety and suicidal tendencies is higher among migraine patients, especially chronic migraineurs [55].

There is a bidirectional relationship between migraine and anxiety disorders, with one increasing the risk of the other. There are three types of anxiety disorders most often associated with migraine: panic disorder (PD), generalized anxiety disorder (GAD), and obsessive compulsive disorder (OCD) [56]. Animal models were used to investigate the relationship between migraine and anxiety. Studies on the cognitive aspects of headaches in animals have been limited. It is possible to assess pain in different aspects with the help of behavioral tests, including sensory-discrimination, affective-emotional, and cognitive tests [57].

The premonitory phase of migraine includes the activation of the posterior and lateral hypothalamus and midbrain ventral tegmentum. Homeostasis alterations that trigger migraines and some of the symptoms during this phase can be explained by the limbic system’s connections [58].

Some current data regarding pathogenesis of anxiety disorders are related to a “threat circuit” in the brain represented by connections between dorsomedial prefrontal cortex, insula, and amygdala. Anxiety disorders are associated with an increased activation of this circuit in response to threatening stimuli [59]. Rodents’ emotionality was initially measured with the open-field test. Rats have an aversion to novel, brightly illuminated, open environments, which is the basis of the test. Anxiety is evaluated primarily using the percentage of inner zone distance (ID%) and the percentage of inner zone time (IT%). In anxious rats, ID% and IT% are lower because they are afraid of exploring and prefer to stay in a safe place, such as the outer perimeter of the open field. According to Bogdanov et al., an open field test was used to assess the correlation between susceptibility to CSD, the most common cause of migraine aura, and anxiety, and they concluded that increased anxiety-like behavior was correlated with a higher frequency of CSD [60].

The limbic system structures are highly sensitive in a patient with chronic migraine because the neuronal hyperexcitability occurring in these areas and neighboring regions causes them to be highly sensitive. It is likely that this sensitization is responsible for the provocation of anxiety disorders when confronted with noxious stimuli [61].

Migraines and anxiety disorders are also often associated with dysfunctions of the serotonergic system. Serotonin (5-HT) metabolism can be altered in migraine patients as well as anxiety patients who have serotonin transporter gene polymorphisms [61,62].

By involving the serotonergic system, the microbiota and gut–brain axis can cause both migraine and depression to coexist. There are several ways in which the gut microbiota can affect brain function, including modulating serotonergic, noradrenergic, dopaminergic, glutamatergic, and GABAergic neurotransmission in the brain [63]. The gut microbiota also contains enzymes that regulate the metabolism of tryptophan, which leads to the production of serotonin, kynurenine, or indole derivatives. The microbiota can influence the brain’s production of serotonin [64].

Studies in mice have shown that probiotics reduced anxiety- and depressive-like behavior. Dopamine and serotonin levels in the striatum were significantly increased after chronic Lactobacillus plantarum administration [65]. Lactobacillus brevis non-live strain application stimulated serotonin receptors in intestinal cells, suggesting non-viable microorganisms may also modulate gut–brain axis [66].

An epidemiologic study in France has concluded that people with migraine and anxiety have a lower quality of life, as well as having a higher level of disability when they suffer from comorbid anxiety. They found that 50.6% of subjects with active migraine had anxiety (28.0%) or depression (3.5%) or both (19.1%). There was a lower level of satisfaction with acute treatment among migraine patients with comorbid anxiety than among migraine patients without comorbid anxiety and depression [67].

The relationship between migraine and depression was studied with the help of neurotransmitters and receptors. These two disorders are linked by the imbalance of serotonin (5-HT) neurotransmitters, as evidenced by the response to 5-HT regulators like triptans and selective 5-HT reuptake inhibitors (SSRIs) [68]. Depression has been associated with the monoamine neurotransmitter hypothesis, where there is a decrease in the concentration of monoamine transmitters. Although migraine and depression are linked to dysfunction of monoamine transmitters, the specific mechanism remains unclear. 5-HT levels in migraine patients were found to be chronically low [68,69].

A link exists between migraines and mood disorders in relation to estrogens and their receptors in the hypothalamus. In humans, modulation of the hypothalamic–pituitary–adrenal (HPA) axis and limbic system has been linked to mood disorders and migraine [70]. When administered before menstruation, cutaneous estrogen supplementation, such as the patch or gel, has been shown to reduce migraine symptoms and regulate migraine activity [71]. In the available research, estrogen has been shown to be pharmacologically effective in reducing depression symptoms [72].

The pivotal role of CGRP in migraine’s pathology has been previously discussed. There are studies that found a reduction in depressive symptoms in migraine patients after 3 months of treatment with anti-CGRP medication [73]. In spite of the exhaustive study of CGRP’s vasodilation and inflammation activity in migraine, its role in depressive-like behaviors has remained unclear. A CGRP infusion performed intracerebroventricularly induces anxiety behaviors and improves learning and memory. Researchers have found that patients with depression have altered levels of CGRP [74,75]

It has been found that patients with chronic migraine suffer from significantly elevated serum CGRP levels, even when they do not have migraine attacks [76]. Monoclonal anti-CGRP (ligand or receptor) antibodies reduce the severity of depressive symptoms in migraine patients, regardless of migraine reduction. The improvement of depressive symptoms can be a sign that erenumab is providing treatment benefits in migraine [73].

In addition to their shared genetic background, depression and migraine share genes from the serotoninergic, dopaminergic, and GABAergic systems, as well as variants of the MTHFR (methylenetetrahydrofolate reductase) and BDNF (brain-derived neurotrophic factor) genes. However, the characterization of migraine patients with comorbid depression requires larger studies [77].

Several experimental studies have shown that mitochondrial dysfunction and inflammation contribute to depression [78]. In some migraine studies, mitochondrial DNA has been implicated as playing an important role, but additional studies involving full mitochondrial genome sequencing are needed [79].

The presence of comorbid depression should be considered when determining a course of treatment, since certain drugs used for prevention of migraine such as topiramate, flunarizine, and beta-blockers can exacerbate depression symptoms, whereas valproate may have mood-stabilizing effects [80]. Studies suggest that depression or depressive symptoms are associated with a poorer response to anti-CGRP monoclonal antibodies, although not all studies have found this connection [81,82,83].

## 6. Migraine, the Gut–Brain Axis and Irritable Bowel Syndrome

As the name suggests, the gut–brain axis refers to the bidirectional communication between the gut and the brain, which integrates immunological, neural, and hormonal signals [84]. The microbiota influences many systems and organs in the human body, including the brain. In migraine pathophysiology, the gut–brain axis plays an important role and gut microbiota are crucial in modulating this link, but the exact mechanisms are unclear [85,86]. This connection suggests that modulating the microbiota could play a significant role in migraine treatment and represents a promising research area [85].

Lanza et al. and Kang et al. found preclinical evidence of microbiota involvement in migraine in animal models. A study by Kang et al. shows that gut microbiomes play a vital role in mechanical pain sensation and migraine pathogenesis. A study by these authors found increased basal mechanical sensitivity, as well as no further hyperalgesia induced by NTG (Nitroglycerine) administration. By restoring the gut microbiota, basal mechanical hyperalgesia and hyporesponsiveness to NTG were reversed. Additionally, fecal microbiome transplantation (FMT) successfully reproduced migraine-like pain susceptibility in mice. Lanza M et al. demonstrate that SCFAs play a significant role in migraine pathogenesis, as well as in microbiota composition and intestinal permeability. As a result of NTG-induced migraine in mice, sodium propionate and sodium butyrate significantly restored intestinal permeability and integrity, as well as the composition of the intestinal microbiota. Through bacterial metabolism of dietary fiber, sodium butyrate (SB) and sodium propionate (SP) are naturally produced in the body. Through the promotion of a healthy gut microbiota, SB and SP may play a role in the prevention of migraines [87,88].

There are many factors that contribute to migraine attacks, including proinflammatory factors, gut microbiome composition, neuropeptides, serotonin pathways, stress hormones, and dietary factors [89].

The gut microbiota, which includes Bacteroides and Firmicutes, is a complex, dynamic system influenced by diet, lifestyle, infections, antibiotic therapy, and hormones [85]. In addition, early life experiences, such as delivery and breastfeeding, are crucial for the development of this complex system [89]. Vagal nerves, tryptophan metabolites, and short-chain fatty acids (SCFAs) act as links between microbiota and the brain [90]. The metabolism and neurotransmission of migraine patients may be altered compared to those without migraine [86].

The gut–brain axis can be affected by dysbiosis, which can contribute to the chronicity of migraine pain by up-regulating TNF-α level in the trigeminal nociceptive system [91]. Microbial dysbiosis may be related to diet, alcohol dependence, smoking, obesity, proton pump inhibitors, selective serotonin reuptake inhibitors, and antibiotic therapy [85].

There are bacteria associated with a healthy microbiome that produce short-chain fatty acids (SCFAs, namely: butyrate, propionate, and acetoacetate), while those that are considered negative in the microbiome are potential pathogens and produce bacterial toxins such as lipopolysaccharides (LPS) [92].

Microbiota may indirectly regulate primary sensory neurons in the dorsal root ganglia’s neuronal excitability by activating nonneuronal cells to release proinflammatory cytokines (such as TNF-a, IL-1b, and IL-6), chemokines (such as CCL2 and CXCL1), or anti-inflammatory cytokines (such as IL-4). These mediators trigger inflammation-induced hypernociception. The cytokines and chemokines secreted by microglia and astrocytes affect synaptic neurotransmission by increasing glutamate and decreasing gamma-amino-butyric acid (GABA), resulting in pain and hypersensitivity [84].

The production of short fatty acids (SCFAs) and the composition of the microbiota are directly related [82]. This link is related to the free fatty acid receptor 3 (FFAR3) expressed in the vagal nerve fibers as part of the short-chain fatty acid signaling pathway [93].

The CGRP pathway is also considered to be a modulation pathway. During dysbiosis, an increase in the production of the calcitonin gene-related peptide (CGRP) is caused, which may be linked to migraines. The role of microbiota in CGRP-mediated pain signaling, however, would require further research into individual agonists and different subtypes of receptors [94].

As well as influencing the metabolism of neurotransmitters, microbiota may also produce neuroactive substances. Bacteria such as Candida, Escherichia coli, Enterococcus, and Streptococcus produce serotonin. Bacillus and Serratia produce dopamine, while Escherichia and Saccharomyces produce norepinephrine. Bifidobacterium and Lactobacillus generate GABA. Neurotransmitters produced in the gut did not reach the brain with one exception (GABA, as GABA transporters are present in the blood–brain barrier). Despite this, intestinal neurotransmitters may indirectly affect the brain by acting on the enteric nervous system [90].

The presence of chronic pain negatively impacts the quality of life for millions of people worldwide. The most effective treatment method for migraines is medication, but alternative methods are also available. Targeting the gut microbiota seems to be a promising therapeutic strategy for the treatment of pain [89].

Recent preclinical data indicating microbiota involvement in animal models of migraine and their implications for humans must be considered as part of the bench-to-bedside interrelationships and reciprocal effects between migraines and microbiota. Many studies have reported a link between diet triggers and migraine [95]. It is evidence that dietary compounds may modulate CGRP secretion. CGRP also may suppress food intake via induction of anorexigenic neuropeptides, such as cholecystokinin, or inhibiting orexigenic neuropeptides, such as neuropeptide Y [96].

It has been demonstrated that probiotics have immense potential for modulating the gut microbiota and restoring microbial balance. Prebiotics can supplement or replace probiotics and may be beneficial in chronic pain management [84]. The use of probiotics has an impact on the production of SCFA. By suppressing kappa factor B, they enhance epithelium integrity and reduce proinflammatory cytokine levels. Several studies have shown that some prebiotics may be able to drive the proliferation of certain bacterial groups, but they do not have the ability to increase the diversity of microbiomes [85].

Restoring gut bacteria through fecal microbiota transplantation (FMT) is currently being studied in more than 340 clinical trials [85]. Its therapeutic effects are mediated by direct competition between pathogenic bacteria and commensal microbiota, protection of the intestinal barrier, and stimulation of the intestinal immune system. However, this is an invasive procedure and FDA underlines the risk of infection transmission [84].

According to symptomatically based criteria, many IBS (irritable bowel syndrome) subjects also suffer from migraine with an estimated odds ratio of 2.66 [97]. It has been suggested that defective serotonin could explain the pathophysiology of IBS and migraine. Serotonin regulates gut motility, secretion, and sensation, as well as being an essential central nervous system neurotransmitter [98]. In IBS, defective 5-HT activity has been found to contribute to the pathogenesis and for this reason, agonists and antagonists are used to treat IBS. In a similar vein, 5-HT1B/1D agonists, in particular sumatriptan (5-HT1B/1D agonist), have been used for treating migraine headaches for a long time [97,99]. There is also evidence that cannabinoids reduce the activity of trigeminovascular vessels and improve gastrointestinal function, hence making them ideal for the treatment of migraine, IBS [99].

There is growing evidence that management of intestinal function may be beneficial for migraine patients in other studies, where diets based on IgG elimination are combined with probiotics. In the gut–brain axis, serotonin is a key factor, and enteric bacteria control its production. Probiotics may relieve migraines by interfering with the intestinal barrier in this way [100].

## 7. Conclusions

A variety of pathophysiological mechanisms can explain the association of migraine with other disorders such as epilepsy, anxiety, depression, and disorders of the microbiota.

Identifying these comorbidities and the underlying mechanisms may improve treatment options for subgroups. Comorbidities in migraine may decrease quality of life, and treating these disorders may improve pain recurrence. Obtaining a complete medical history can reveal migraine triggers and comorbidities, which can be treated to reduce migraine attacks. In this article we described a few common links between migraine and some comorbid conditions, and we suggest some treatment options for migraine in consideration of these common pathways that link migraine with these other health conditions. It is becoming more and more evident that the gut–brain axis may hold the key to treating central nervous system diseases. The last consideration to be taken into account is that migraine comorbidities may also be comorbid with one another, which might impact pathophysiological mechanisms and treatment options. The treatment of migraines simultaneously with comorbid disorders should be conducted in a dedicated trial.

## Data Availability

The data are available in the cited sources.

## Abbreviations

AMPA	a-amino-3-hydroxy-5-methyl-4-isoxazolepropionic acid
ASD	anti-seizure drugs
BDNF	brain-derived neurotrophic factor
CGRP	calcitonin gene-related peptide
CSD	cortical spreading depression
FDA	Food and Drug Administration
FFAR3	free fatty acid receptor 3
FHM	familial hemiplegic migraine
FMT	fecal microbiota transplantation
GABA	gamma-amino-butyric acid
GAD	generalized anxiety disorder
HPA	hypothalamic-pituitary-adrenal axis
5-HT	5-hydroxytryptamine
IBS	irritable bowel syndrome
LPS	lipopolysaccharides
MTHFR	methylenetetrahydrofolate reductase
NTG	Nitroglycerine
NMDA	N-methyl-D-aspartate
OCD	obsessive compulsive disorder
PACAP	adenylate cyclase activating peptides
PD	panic disorder
SCFAs	short-chain fatty acids
SSN	superior salivatory nucleus
SSRIs	selective 5-HT reuptake inhibitors
